# Culture and Unmerited Authorship Credit: Who Wants It and Why?

**DOI:** 10.3389/fpsyg.2016.02017

**Published:** 2016-12-27

**Authors:** Xiaopeng Ren, Hong Su, Kewen Lu, Xiawei Dong, Zhengzheng Ouyang, Thomas Talhelm

**Affiliations:** ^1^CAS Key Laboratory of Behavioral Science, Institute of PsychologyBeijing, China; ^2^University of Chinese Academy of SciencesBeijing, China; ^3^National Science LibraryBeijing, China; ^4^Booth School of Business, University of ChicagoChicago, IL, USA

**Keywords:** culture, unmerited authorship, science, collectivism, individualism

## Abstract

Unmerited authorship is a practice common to many countries around the world, but are there systematic cultural differences in the practice? We tested whether scientists from collectivistic countries are more likely to add unmerited coauthors than scientists from individualistic countries. We analyzed archival data from top scientific journals (Study 1) and found that national collectivism predicted the number of authors, which might suggest more unmerited authors. Next, we found that collectivistic scientists were more likely to add unmerited coauthors than individualistic scientists, both between cultures (Studies 2–3) and within cultures (Study 4). Finally, we found that priming people with collectivistic self-construal primes made them more likely to endorse questionable authorship attitudes (Study 5). These findings show that culture collectivism is related to unmerited authorship.

## Introduction

When submitting a paper to an academic journal, researchers usually have to decide whether to include the coworker, who provided only minor contribution to their work such as supplying a few comments, on the author list. But what are the criteria in the real life setting, do all researchers interpret the criteria equally and why? Is there a cultural difference? Imagine two researchers, James from individualistic society and Baohong from collectivistic society, facing the above described scenario. Will they behave similarly in a given situation or completely different? Although there is an official standard in scientific communities, adhering to it strictly would probably cause some distress among colleagues. Cross-cultural studies found that individuals from collectivist cultures are more likely to try to avoid offending others than those from individualist cultures (Lee et al., 2000). In scientific community, this would imply that collectivistic scientists are more likely than their individualistic counterparts to violate the authorship criteria in favor of their colleagues. Nevertheless, according to our knowledge this idea still has not been tested directly. Therefore, in this study we ask whether there are any cultural differences behind James and Baohong's decisions. We will argue that James is more likely to oppose adding the coworker on the authorship's list, while Baohong is more likely to accept it.

## What is unmerited authorship?

Most academic journals or scientific associations have their own guidelines and criteria for assigning authorship credit (International Committee of Medical Journal Editors, 2008; Marcovitch and Committee on Publication Ethics, 2009). Unmerited authorship includes honorary authorship, gift authorship, and guest authorship, and researchers have argued that it is more prevalent than other types of misconduct in the scientific community across almost all disciplines and regions (Martinson et al., 2005; Apgar and Congress, 2006; Tryon et al., 2007; Smith et al., 2014). In one survey, Marusic and colleagues found that 29% of respondents misused authorship at some time during their scientific career. This rate was 10 times higher than rates of fabrication, falsification, or data modification. According to a recent meta-analysis, these other types of misconduct occur at a rate of 2% (Marušic et al., 2011).

Unmerited authorship credit has negative consequences (O'Brien et al., 2009; Wager, 2009). Unmerited authorship dilutes the contribution of the actual authors, and it separates the reward mechanisms of science (publications) from the objective input (research work). Some argue that it reinforces the abuse of hierarchy in academia because it often rewards established, senior researchers (O'Brien et al., 2009). Others argue that it misinforms readers about who did the work (Wager, 2009).

If unmerited authorship has negative consequences, why does people do it? Researchers have discussed many factors, such as the incentives created by the system of rewarding researchers for publications in the scientific community (Claxton, 2005). Researchers have also pointed to feelings of obligation, long-term cooperation, team responsibility, and power relations (Mixon Jr and Sawyer, 2005). In this study, we test whether culture plays a role.

## Are there cultural differences in unmerited authorship?

Although researchers have studied unmerited authorship, we know very little about whether there are national differences. Scientific communities often rely on teamwork, but the way people treat each member's contribution may differ significantly across cultures. For instance, should people be added on the author list if their contribution was limited to offering helpful advice or a suggestion on a draft of the paper? Even though there are defined principles and procedures for determining authorship (International Committee of Medical Journal Editors, 2008), we do not know how well authors apply these procedures in their daily practice (Fetters and Elwyn, 1997; Marušic et al., 2011).

There is no direct evidence that unmerited authorship is related to individualism/collectivism, but some previous results hint at it. For example, Fetters and Elwyn (1997) argued that culture may influence how people perceive contributions. They analyzed English-language Japanese and US journals and found that Japanese papers included 2–3 more authors on average than US journals, even controlling for journal quality. We do not know whether that higher number includes more unmerited authors or simply a more collaborative culture.

Another hint came from a meta-analysis of 14 studies of authorship problems (such as adding a co-author who did little or no work) in the US, the UK, and journals from other countries (Marušic et al., 2011). This study found large regional differences: non-USA/UK journals had a significantly higher proportion of reported authorship problems than USA/UK journals (55 vs. 23%). The researchers argued that this difference cannot be explained by linguistic reasons or differences in formal bodies directing research integrity and economics (Marušic et al., 2011).

If so, it suggests that culture may play an important role. The US and UK are individualistic cultures, whereas the non-US and non-UK cultures in that study were largely collectivistic cultures, such as India and Bangladesh. However, that study did not directly test whether collectivism was systematically related to unmerited authorship. And because that study was not focused on cultural differences, it only loosely compared US/UK vs. “rest of world.”

## Unmerited authorship credit and relationship styles

The results from the survey of authorship problems suggest that it is worth testing whether individualism/collectivism is related to these differences. But what theoretical reasons are there to expect a link between collectivism and unmerited authorship?

### Relationship tightness and indebtedness

Collectivist cultures have tighter relationships and social networks that are based on reciprocal obligations (Markus and Kitayama, 1991). For example, studies have found that people in China and Japan people feel more indebted to others after receiving a favor (Hitokoto, 2016; Oishi and Komiya, 2016). This emphasis on repaying favors could mean that people in collectivistic cultures are more likely to feel stronger feelings of indebtedness to collaborators and repay them with authorship, even if those contributions were for previous projects or other unsuccessful projects.

### Avoiding offending other people

Researchers have also found evidence that people in collectivistic cultures are more focused on avoiding offending other people (Yamagishi et al., 2008). For example, researchers offered participants in Japan and the United States a pen as a reward for a study (Kim and Markus, 1999). Participants could choose between three blue pens and one red pen (or three red pens and one blue pen). People in Japan were less likely to choose the unique pen—unless they were told they were the last participant in the study. This suggests that Japanese participants were concerned about offending other people if they took the last available pen of a certain color. Furthermore, in a cross-cultural studies on promotion vs. prevention regulatory focus (Lee et al., 2000), participants were asked to judge the importance and feelings of the players in tennis tournament final match in promotion vs. prevention framework(win or lose the match). Hong Kong Chinese are more prone to adopt avoid failure strategy (prevention regulatory focus) than Americans, especially in situational and chronic self-construal consistent condition and feel more relaxation and/or agitation emotions (prevention-focused emotion).

### Relational mobility

Cultural differences in relational mobility may also help explain how worried people are about upsetting relationships. Collectivistic cultures like Japan have low relational mobility, meaning it is harder to leave unsatisfying relationships and create new ones (Schug et al., 2010). In contrast, individualistic cultures like the United States operate more like free markets, where it is easy to meet new people and exit unsatisfying relationships.

Thus, in the US, the consequences of offending a collaborator are smaller. And there are many more potential collaborators out there if one particular relationship goes sour (Wang et al., 2011). But in a low-mobility environment like Japan, the costs of offending a collaborator are higher.

### Relationship weight in moral decision-making

Studies comparing moral decision-making across cultures have found evidence that people in collectivistic cultures place more importance on relationships (Miller and Bersoff, 1992; Graham et al., 2009; Yilmaz et al., 2016). Some studies have forced participants to confront a tradeoff between abstract rules vs. relationship duties. For example, one study asked people in India and the US about a man who is taking a ring to his friend's wedding but loses his train ticket (Miller and Bersoff, 1992). After exhausting all other options, participants have to decide whether it is better to steal a train ticket from a rich man in order to deliver the ring or miss the train and fail in their duty to the friend. Participants in India were more likely to choose to fulfill the relationship duty; Americans were more likely to uphold the principle that stealing is wrong.

In another study, researchers asked 34,000 participants from North America to Europe, Asia, Africa, and the Middle East to rate how important different moral foundations were to them (Graham et al., 2009). Participants in East and South Asia were more likely to endorse items that stressed loyalty to the in-group than were American participants. Thus, cross-cultural studies give some evidence that people in collectivistic cultures place more importance on relationships in their moral decisions.

With that in mind, consider two researchers in the same lab. A researcher sends out a draft of a paper to the lab group. A team member sends the draft back with a few comments. Should that lab member now join the author list? The official guidelines say no, but maybe it would offend that person. Is it worth upholding the abstract standard and risk offending the other person? If people in collectivistic cultures place a more weight on avoiding offense, scientists from those may be more likely to add the coworker to avoid conflict in relationships.

## Overview of the present study

This study tests for cultural differences in unmerited authorship credit and whether collectivism can explain these differences. We use a multi-method approach to test whether individualism can explain the number of coauthors per paper in the natural sciences. In Study 1, we analyze how many authors per paper different nations have in three top journals (*Cell, Nature*, and *Science*). Of course, having lots of authors on a paper is not always a sign of unmerited authorship. But it is suggestive, and it lays the ground for more direct tests in Studies 2–5.

In Study 2, we test whether nation-level collectivism can explain differences in coauthor practices. In Study 3, we test for differences in authorship practices and collectivism between Chinese and Danish scientists. In Study 4, we test for this relationship at the individual level among a larger sample of Chinese scientists. In Study 5, test for causality by priming people with individualism or collectivism and then measuring their co-authorship preferences.

## Study 1

In Study 1, we test whether individualism is related to the number of co-authors using archival data on individualism and co-authorship. We use the number of authors per paper as a number that might suggest unmerited authorship. Having many authors does not prove unmerited authorship. It is also entirely plausible that people in collectivistic cultures are more likely to work in teams.

However, long author lists are also consistent with unmerited authorship (Geelhoed et al., 2007). If unmerited authorship is more common in collectivistic cultures, we should expect that papers from collectivistic cultures have more authors on average. Thus, this finding would at least be consistent with the hypothesis that unmerited authorship is more common in collectivistic cultures and thereby merit further research.

Despite the inferential leap, there is indirect evidence to support the idea that long author lists are more likely to contain unmerited authors. For instance, several authorship guidelines suggest that scientists who contribute less than 10% to the paper should not be included on author list, but rather in the acknowledgments section (International Committee of Medical Journal Editors, 2008). Add to that, a survey found that authors in the fourth position and greater usually contribute less than 10% to the paper (Geelhoed et al., 2007). If that phenomenon is generally true, longer author lists (particularly over 4) suggest unmerited authorship.

Having to make inferences like these is one of the limits of using real-world archival data. However, the benefit is that archival data like this reflects real-world behavior. For these reasons, the results of Study 1 are suggestive but not conclusive. We use more direct measures of authorship malpractice in Studies 2–5.

### Methods

#### Number of coauthors per paper

We obtained authorship data from the Web of Science 2002–2011. Because this study focuses on the natural sciences, we selected three top journals (*Cell, Nature*, and *Science*) because they are influential and represent many fields in the natural sciences. We first categorized papers to nations based on the first author/corresponding author. A few papers had different first authors and corresponding authors who were in different countries. We categorized those papers based on the corresponding author. We only kept the 21 most-represented nations because the remaining nations had fewer than 100 papers, which would make estimates of co-authors unstable (Table 1).

**Table 1:** **Descriptive statistics of authors in CNS of the top 21 nations 2002–2011**.

**Nation**	**Number of papers**	**Mean # authors**	**Median # authors**
USA	22,811	4.85	3
England	4105	4.04	2
Germany	2118	5.73	4
France	1228	6.32	4
Canada	1148	4.64	2
Japan	1086	10.02	7
Switzerland	882	4.57	3
Netherlands	737	4.71	3
Australia	676	4.20	2
Italy	457	7.23	4
Scotland	456	3.78	2
China	394	8.08	5
Israel	328	3.87	3
Sweden	309	5.78	3
Spain	295	5.38	3
Denmark	236	6.28	4
Austria	224	5.61	4
Belgium	175	6.48	3
Norway	128	4.15	2
India	106	2.52	1
South Korea	105	8.57	8

#### Individualism

We used Hofstede and Hofstede's (2001) data as a nation-level index of individualism. We also collected variables such as GDP per capita and Gini Coefficient as control variables. The GDP per capita of nations was available from the (United Nations Development Program, 2011, http://hdr.undp.org/en/media/HDR_2011_Statistical_Tables.xls). We obtained Gini Index percentage points ranging from 0% (completely equal income) to 100% (completely unequal) from the World Institute for Development Economics Research of United Nation University (United Nations University-World Institute for Development Economics Research, 2008, http://www.wider.unu.edu/research/Database/en_GB/wiid/). Research and development expenditure data came from the Eurostat database (Eurostat, 2010, http://epp.eurostat.ec.europa.eu/statistics_explained/index.php/R_%26_D_expenditure) and the OECD database (OECD, 2010, http://www.oecd-ilibrary.org/science-and-technology/gross-domestic-expenditure-on-r-d_2075843x-table 1), except for the data for India, which came from the UN Science Report 2010.

### Results

We examined zero-order correlations between individualism and (1) the mean number of co-authors per paper and (2) the median number of co-authors per paper (Table 2). Individualism was negatively correlated with the mean number of co-authors per paper, *r*_(21)_ = −0.529, *p* < 0.05, and the median number of co-authors per paper, *r*_(21)_ = −0.651, *p* < 0.01 (Table 2).

**Table 2:** **Descriptive statistics and zero-order correlation of individualism and number of co-authors**.

	***M* ± SD**	**1**	**2**	**3**	**4**
1 GDP	31.332 ± 10.074				
2 Gini coefficient	32.71 ± 4.55	−0.521^*^			
3 R&D expenditures	2.27 ± 0.90	0.329	−0.230		
4 Individualism	65.81 ± 20.73	0.594^**^	−0.170	−0.129	
5 Mean co-authors	5.56 ± 1.80	−0.087	−0.018	0.230	−0.529^*^
6 Median co-authors	3.43 ± 1.66	−0.076	−0.035	0.407	−0.651^**^

*GDP, GDP per capita; R&D, gross domestic expenditure on R&D 2002–2011*;

* *p < 0.05*,

** *p < 0.01*.

**Figure 1:**
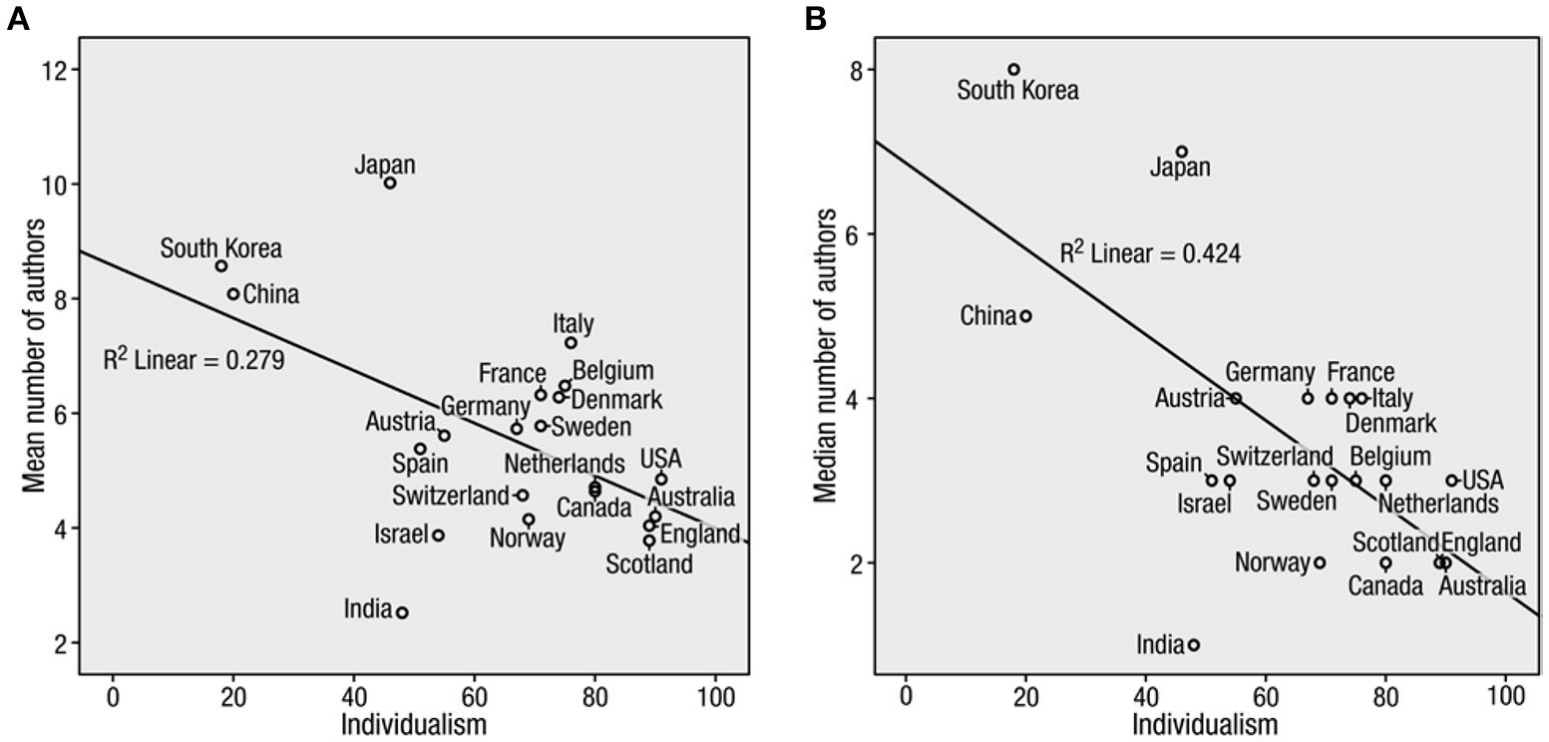
**Correlation between Hofstede's individualism score and number of authors per paper (Study1)**. Scatter plots (with best-fitting regression lines) show results for mean number of authors **(A)** and median number of authors **(B)**.

Next, we ran a fixed-effects regression controlling for economic variables such as GDP per capita, Gini coefficients, and gross domestic expenditure on R&D. Controlling for these variables, individualism was still negatively correlated with the average number of co-authors per paper [*B*_(21)_ = −0.064, *SE* = 0.024, β_(21)_ = −0.737, *t*_(21)_ = −2.634, *p* = 0.018, 0.001, 95% CI = [−0.115, −0.012]] and the median number of co-authors per paper [*B*_(21)_ = −0.069, *SE* = 0.018, β_(21)_ = −0.865, *t*_(21)_ = −3.905, *p* = 0.001, 95% CI = [−0.107, −0.032]]. In fact, the standardized coefficients for individualism were larger after controlling for economic variables. Thus, evidence from the 21 most-represented nations in CNS showed that individualistic countries have fewer co-authors per paper (Figure 1).

Even though these results fit with our initial premise that collectivistic cultures would have more co-authors, this study has several limitations. First, we cannot be sure that large author lists contain more unmerited authors. Second, we cannot infer the psychological motivations of the authors. Finally, the data are correlational and therefore cannot prove causality.

## Study 2

In Study 2, we sent surveys to the first/corresponding authors of papers in *Cell, Nature*, and *Science* from 21 nations with the most publications. We tested whether the individualism of their culture would predict their beliefs and intentions about adding coauthors.

### Methods

#### Participants

We emailed the first/corresponding authors of the papers in Study 1 and invited them to fill out a survey. For nations that had fewer than 1000 authors (such as South Korea), we invited all of the authors to fill out the survey. For nations with more than 1000 authors (such as the US), we chose 1000 authors at random. We contacted a total of 11,508 first and corresponding authors.

We received 322 complete responses for a response rate of 2.8%. This response rate is low, but may not be surprising because top scientists are busy, with lots of demands on their time. Furthermore, this response rate is similar to a previous survey of *Nature* and *Science* authors (Li et al., 2011).

A total of 25 participants worked in collectivistic cultures (17 China, 4 India, 4 Japan, 1 Singapore), and 286 worked in individualistic cultures (87 US, 63 UK, 21 France, 20 Australia, 15 Canada, 14 Germany, 13 Italy, 11 Netherlands, 39 other). There were also 16 scientists who did not report their nationality and consequently were not included.

There were also two scientists who worked in Eastern cultures but were originally born in Western cultures, as well as 24 scientists who worked in Western cultures but were born in Eastern cultures^1^. We excluded them from analysis. The final analysis included 263 scientists from individualistic cultures (226 men, 37 women; mean age = 48.21) and 23 from collectivist cultures (20 men, 3 women; mean age = 45.61).

#### Procedure

Participants completed the survey online on a Qualtrics page hosted at the Department of Psychology at the University of Michigan (https://umpsych.qualtrics.com). Participants gave consent before beginning the questionnaire.

#### Measures

We developed a 6-item scale to measure co-author practices. Items asked about with unsuitable behaviors initiated by the respondent (such as, “I have included a colleague as a co-author who made no contribution to the manuscript, but with whom I have a personal relationship”; see Supplementary Material Table 1A for all items) or initiated by their colleagues (for example, “I have been included as a co-author on a manuscript to which I made no contribution, but with whose author I have a personal relationship”). The Cronbach's alpha of this scale in this sample was 0.75. Participants rated each item on a scale from 1 (*never*) to 4 (*often*).

### Results

We ran independent sample *t*-tests with gender as a between-subject factor and coauthor practices as dependent variables, showing no significant gender effect, *t*_(284)_ = 0.26, *p* = 0.80. Men (mean = 1.36, SD = 0.36) and women (mean = 1.37, SD = 0.41) had similar coauthor attitudes.

**Figure 2:**
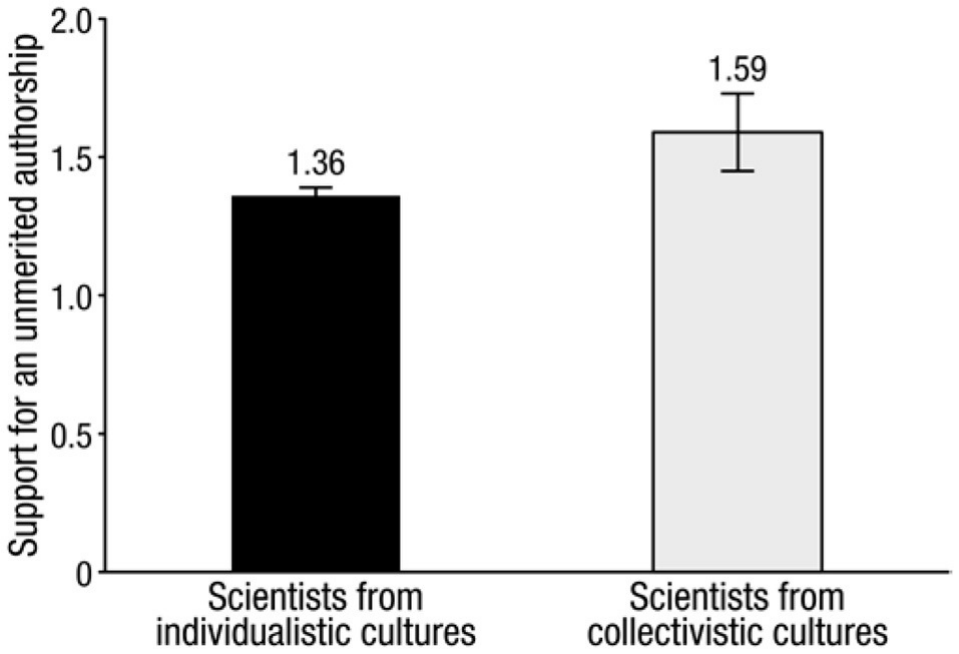
**Scientists from individualistic cultures (left) were less likely to endorse unmerited authorship practices than scientists from collectivistic cultures (right)**. Bars = 1 standard error of the mean.

We ran an independent sample *t*-test with culture as a between-subject factor and coauthor practices as the dependent variable. The effect of culture was significant, *t*_(285)_ = 3.21, *p* = 0.001, *d* = 0.52. Individualistic scientists (mean = 1.59, SD = 0.53) were more reluctant than collectivistic scientists (mean = 1.36, SD = 0.34) to engage in unmerited co-authorship practices (Figure 2).

The results showed that authors from individualistic cultures were more likely to endorse academic community standards on authorship. Authors from collectivistic cultures were more likely to say they have added authors who did not meet standards for authorship. The results also showed that scientists from collectivistic cultures who are currently working in an individualistic culture had stricter attitudes about unmerited authorship than scientists not working in an individualistic culture. This suggests that scientists are enculturating to the norms of the new culture (or perhaps selecting into these cultures).

Study 2 improved on Study 1 because it measured unmerited authorship beliefs directly. However, one weakness is that Study 2 was based on participants' subjective feedback, which might be different from their actual behavior.

## Study 3

Studies 1 and 2 had the benefit of measuring actual publishing data and top scientists. However, the surveys were brief email surveys and were thus relatively uncontrolled. In Study 3, we tested Danish and Chinese scientists in a more controlled lab setting.

### Methods

#### Participants

Participants were 16 Chinese graduate students (31.3% female, mean age = 23.88, SD = 2.31) and 16 Danish graduates (68.7% female, mean age = 24.06, SD = 1.34) at the Sino-Danish Center for Education and Research in Beijing^2^. They were all natural sciences majors. They received 20 RMB (US$3.15) for their participation.

#### Materials and procedure

All study materials, instructions, and tasks were prepared both in English and Chinese and then proofread by two translators, including one of the authors. They then worked together to ensure the two versions were identical in meaning.

#### Scenarios

First, participants were asked to write a conclusion to two scenarios we created:

Imagine you are a scientist planning to submit a paper to a scientific journal relevant to your research topic. You are the one who designed the research, conducted the study, and analyzed the data. You are also the one who wrote the paper reporting the results. You have a colleague whom you have previously worked with, and whom you plan to work with in the future. However, this colleague has not made any contribution to this study. Your colleague asks if he/she can be included as a co-author.

Scenario 1 is designed to measure willingness to add a colleague as a coauthor in a situation where the colleague helped but did not contribute enough to merit authorship. Scenario 2 is designed to measure willingness to be added by a colleague as a coauthor:

Imagine you are a scientist working in a lab. A colleague from your lab has just finished writing a paper and plans to submit it to a scientific journal. You have collaborated with this colleague in the past, and you plan to do so in the future. Although you had no involvement in the current study or in producing the paper, this colleague offers to add you as a co-author.

Afterwards, they rated how likely they would be to include a colleague as a co-author or accept being included as a co-author on a scale from 1 (*extremely unlikely*) to 7 (*extremely likely*).

Then participants took the 24-item Self-Construal Scale (Singelis, 1994; Kwan et al., 1997). This scale measures the independent self (e.g., “I enjoy being unique and different from others in many aspects”) and the interdependent self (e.g., “I will sacrifice my self-interest for the benefit of the group I am in”). Participants rated the items from 1 (*strongly disagree*) and 7 (*strongly agree*). The scale had high internal consistency (α = 0.73 for independent self and 0.74 for interdependent self). Finally, participants completed demographic items.

### Results

We first tested for differences in individualism between the Chinese and Danish graduate students. We scored individualism as independent self minus interdependent self. In a 2 × 2 ANOVA with nation and gender as between-subject factors, the main effect of nation was significant, *F*_(1, 32)_ = 23.89, *p* = 0.001. The Danish (mean = 0.93, SD = 0.92) were more individualistic than the Chinese (mean = −0.25, SD = 0.47). The main effects of gender and gender-nation interaction were not significant.

Next, we ran a 2 × 2 ANOVA on adding a coworker as a coauthor with nation and gender as between-subject factors. The outcome showed a significant nation effect, *F*_(1, 30)_ = 45.49, *p* < 0.001, $\eta_{p}^{2}$ = 0.62, 95% CI = [1.768, 3.269]. The Danish (mean = 2.50, SD = 1.31) were more reluctant than the Chinese (mean = 5.06, SD = 0.47) to add coworkers as coauthors, *t*_(30)_ = 7.64, *p* < 0.001, *d* = 2.60, 95% CI = [1.878, 3.247]. The main effects of gender and the interaction between gender and nation were not significant.

Looking at the raw numbers, 4 was the midpoint of the scale and represented a neutral attitude. On average, the Danes were well below neutral (2.50), refusing to add the colleague. The Chinese students tended to accept the practice (5.06).

Next, we ran a 2 × 2 ANOVA with nation and gender as between-subject factors and being added by a coworker as a coauthor as the outcome. There was a significant effect of nation, *F*_(1, 32)_ = 14.15, *p* = 0.001, $\eta_{p}^{2}$ = 0.33, 95% CI = [1.062, 3.575]. As predicted, the Danes (mean = 3.00, SD = 1.86) were more reluctant than the Chinese (mean = 5.44, SD = 1.27) to be added as a coauthor, *t*_(30)_ = 4.33, *p* < 0.001, *d* = 1.53, 95% CI = [1.289, 3.586]. The main effects of gender and the gender-nation interaction were not significant.

**Figure 3:**
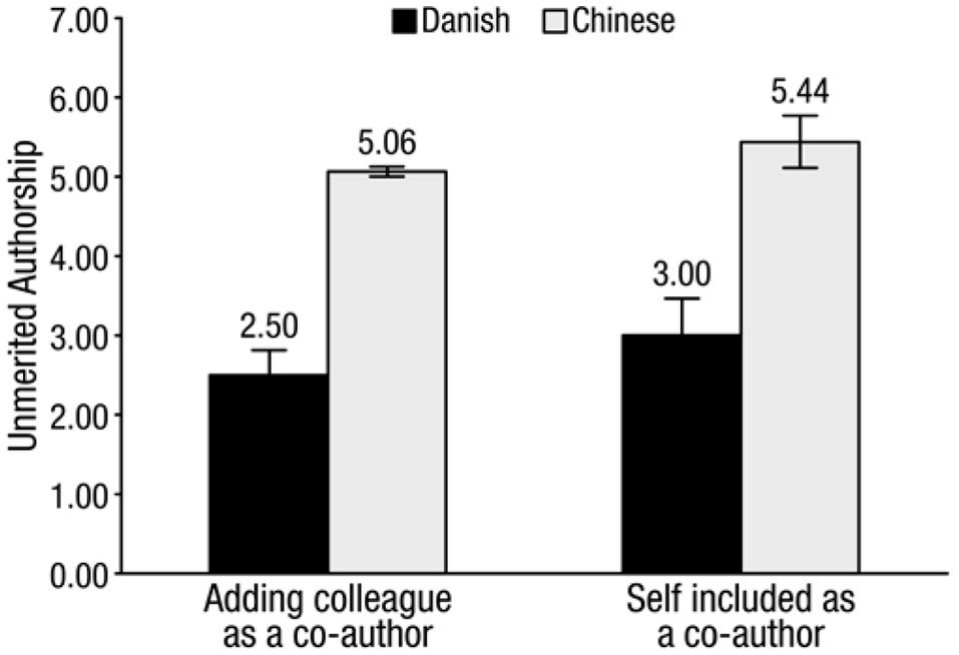
**Tendency to add colleague as coauthor and be added as coauthor by culture**. Bars = 1 standard error of the mean.

As in Scenario 1, the Danish mean (3.00) was below the midpoint, representing refusal. The Chinese mean (5.44) was above the midpoint, representing acceptance. Consistent with the results of Study 1 and Study 2, this laboratory study found that people from the collectivistic culture were more likely to add inappropriate co-workers to the author list than people from the individualistic culture (Figure 3).

This study found evidence that Danish and Chinese scientists have different attitudes about unmerited authorship. Although not all graduate students have published papers, graduate students like these have at least started thinking about questions of authorship and observed how their colleagues and advisors deal with questions of authorship. Their beliefs corresponded to the models practiced by leading scientists in their cultures. This suggests that these scientists have had exposure to their culture's scientific authorship practices.

## Study 4

Study 3 tested for differences between cultures, but it did not test whether individualism was systematically related to authorship attitudes. Study 4 tests this question directly by measuring both individualism and authorship attitudes at the individual level. Study 4 also directly measures publication experiences of participants.

### Methods

#### Participants

Ninety-three Chinese graduate students at the University of the Chinese Academy of Sciences (49.5% female, mean age = 24.58, SD = 1.98) participated in the study; they were all natural sciences majors. The participants received 20 RMB (US$3.15) as compensation.

#### Materials and procedure

All study materials, instructions, and tasks were the same as in Study 3. The only difference was that the materials were in Chinese only. As in Study 3, we calculated individualism as independent self minus interdependent self.

#### Publication experience

We measured publication experience with a yes/no item: “Have you published an academic paper before?”

## Results and discussion

We examined zero-order correlations of individualism and publication experience with (1) adding a colleague as coauthor and (2) being added by a colleague as co-authors. Individualism was negatively correlated with adding a colleague as coauthor, *r*_(93)_ = −0.22, *p* = 0.03, and being added by a colleague as co-authors, *r*_(93)_ = −0.07, *p* = 0.49. Publication experience was negatively correlated with adding a colleague as coauthor, *r*_(93)_ = −0.23, *p* = 0.03, and being added by a colleague as a co-author, *r*_(93)_ = −0.14, *p* = 0.17 (Table 3).

**Table 3:** **Descriptive statistics and correlation of individualism and unmerited authorship**.

	***M* ± SD**	**1**	**2**	**3**	**4**
1 Gender	–				
2 Age	24.58 ± 1.98	−0.03			
3 Publishing experience	1.61 ± 0.49	−0.32^**^	−0.28^**^		
4 Individualism	−0.86 ± 0.72	0.06	−0.02	−0.01	
5 Adding colleague as co-author	3.44 ± 1.38	0.18	0.11	−0.23^*^	−0.22^*^
6 Being added by colleague as co-author	3.87 ± 1.80	0.19	−0.23^*^	−0.14	−0.07

*n = 93*,

* *p < 0.05*,

** *p < 0.01*.

Next, we ran a fixed-effects regression controlling for gender, age, major and publication experience. Controlling for these variables, individualism was still negatively correlated with adding a colleague as coauthor, *B* = −0.52, *SE* = 0.19, β = −0.27, *t*_(93)_ = −2.73, *p* = 0.01, 95% CI = [−0.90, −0.14]. The trend was similar for being added by a colleague as a co-author, but it did not reach significance, *B* = −0.30, *SE* = 0.25, β = −0.12, *t*_(93)_ = −1.20, *p* = 0.24, 95% CI = [−0.70, 0.22] (Table 4).

**Table 4:** **Hierarchical regression predicting unmerited co-authorship**.

**Predictor measures**	**Adding a colleague as co-author**				**Being added by a colleague as a co-author**			
	**Step 1**		**Step 2**		**Step 1**		**Step 2**	
**Δ*R*^2^**	**0.058**		**0.133**		**0.102**		**0.116**	
	***B***	**95%CI**	***B***	**95%CI**	***B***	**95%CI**	***B***	**95%CI**
Gender	0.31	[−0.29, 0.91]	0.34	[−0.24, 0.92]	0.39	[−0.37, 1.15]	0.41	[−0.35, 1.17]
Age	0.05	[−0.11, 0.23]	0.05	[−0.09, 0.19]	−0.24	[−0.44, −0.04]	−0.25	[−0.45, −0.05]
Pub. Exper.	−0.43	[−1.07, 0.21]	−0.41	[−1.03, 0.21]	−0.60	[−1.40, 0.20]	−0.59	[−1.39, 0.21]
Individualism			−0.52	[−0.90, −0.14]			−0.30	[−0.80, 0.20]

Pub. Exper., publish experience n = 93, ^*^p < 0.05, ^**^p < 0.01.

In sum, the results from Study 4 showed that people who score high on individualism are more likely to oppose adding unmerited co-authors. However, the trend was weaker for being added as a co-author by a colleague. However, Study 4 is limited because it relies on self-report measures of individualism. Several studies have found methodological shortcomings with self-report individualism scales (Peng et al., 1997; Heine et al., 2002).

## Study 5

All of the studies thus far have been correlational, which prevents us from being certain about whether collectivism causes people to be more willing to grant unmerited authorship. Thus, in Study 5, we used priming to experimentally manipulate collectivism and test whether it can influence co-author preferences.

### Methods

#### Participants

A total of 220 graduate and undergraduate students attending the Chinese Academy of Sciences and Beijing Forestry University participated in Study 4 (47.1% female, mean age = 22.25, SD = 1.65). They were all natural sciences majors. The participants received 20 RMB (US$3.15) for participating.

#### Self-construal prime

Participants were primed with either an individualist or collectivist self-construal using the scrambled sentence task (Oyserman and Lee, 2008). Each participant had to unscramble 16 scrambled sentences. All 32 items were from Triandis' Individualism-Collectivism Scale (Triandis and Gelfand, 1998). In the individualism priming condition, the primes contained words such as “individual,” “self-contained,” and “independent” (such as “I rely on myself most of the time” and “I rarely rely on others”). In the collectivism priming condition, prime words included “group,” “friendships,” or “together” (such as “I rarely rely on myself” and “I often rely on others”). The priming and other tasks were in Chinese. Afterwards, participants rated the two scenarios and provided demographic information.

#### Dependent variables

The dependent variables were the same as in Studies 3–4: willingness to add another person as a co-author and willingness to be added as a co-author despite lack of merit.

#### Self-construal manipulation check

After finishing the unmerited authorship task, participants were asked to fill out the Singelis' self-construal scales used in Studies 3–4. This allowed us to check whether the manipulation actually changed participants' individualism.

#### Publication experience

We used education attainment (graduate vs. undergraduate) as a proxy for publication experience. In China, graduate students have to publish at least one paper in an academic journal, while undergraduates do not, especially in the natural sciences. Although some graduate students have not yet published a paper, most graduate students have begun to confront questions of authorship and started to enculturate to the authorship attitudes of their advisor and other students. This should be more true of graduate students than undergraduate students in general.

### Results and discussion

#### Manipulation checks

First we tested whether the scrambled sentence task successfully influenced individualism/collectivism. As for Study 3, we calculated individualism as independent self minus interdependent self. Participants in the individualistic condition (mean = −0.57, SD = 0.75) scored higher on individualism than participants in the collectivistic condition (mean = −0.95, SD = 0.75), *t*_(218)_ = 0.52, *p* < 0.01, *d* = 0.51. These results showed that the manipulation was successful.

#### Adding an unmerited co-author

**Figure 4:**
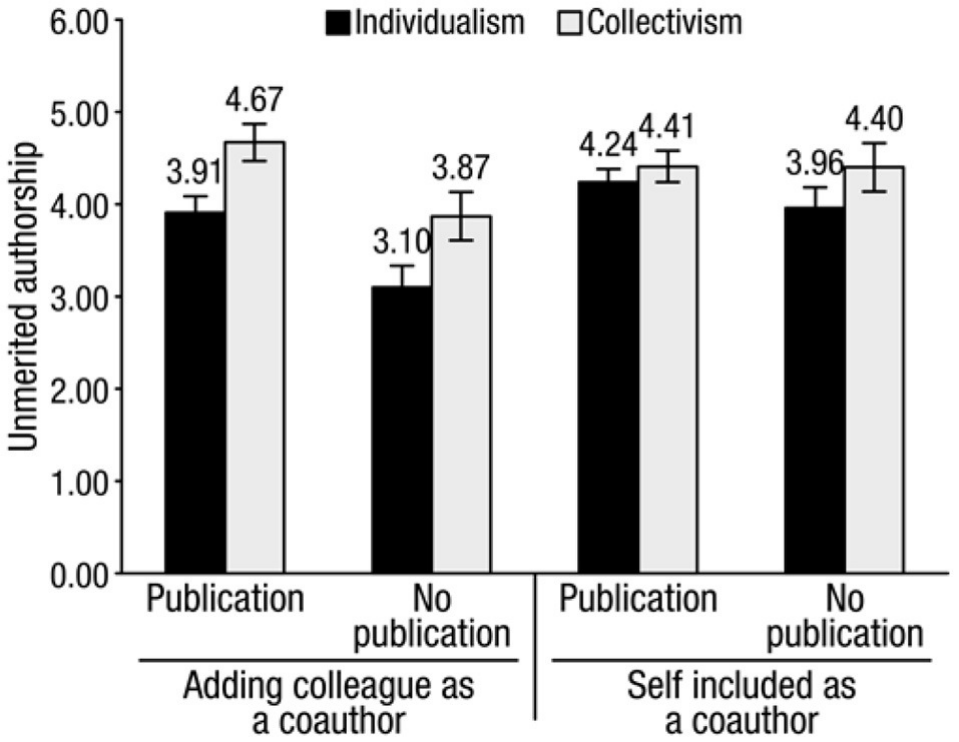
**Willingness to add an unmerited coauthor and to be added as a coauthor by individualism priming and collectivism priming**. Bars = 1 standard error of the mean.

Figure 4 shows the results. We ran a 2 (prime: individualism vs. collectivism) × 2 (publication experience: graduate vs. undergraduate) × 2 (gender: male vs. female) mixed ANOVA on willingness to add a colleague as an unmerited co-author. The main effect of priming was significant, *F*_(1, 219)_ = 9.27, *p* = 0.003, $\eta_{p}^{2}$ = 0.04, 95% CI = [−1.39, −0.05]. Participants primed with individualism (mean = 3.67, SD = 1.46) were less willing to add a coworker than people primed with collectivism (mean = 4.47, SD = 1.39), *t*_(218)_ = −4.24, *p* < 0.01, *d* = 0.57, 95% CI = [−1.56, −0.34].

The main effect of experience publishing was also significant, *F*_(1, 219)_ = 5.46, *p* = 0.02, $\eta_{p}^{2}$ = 0.03, 95% CI = [0.45, 1.86]. Graduate students (mean = 4.34, SD = 1.45) were more willing to add a coworker than undergraduates (mean = 3.48, SD = 1.30), *t*_(218)_ = 4.04, *p* < 0.01, *d* = 0.61, 95% CI = [0.11, 1.34]. There were no other main effects or interaction effects.

#### Being added as a co-author

We ran a 2 (prime: individualism vs. collectivism) × 2 (publication experience: graduate vs. undergraduate) × 2 (gender: male vs. female) mixed analysis of variance on willingness to be added as a coauthor. The main effect of priming was not significant, *F*_(1, 219)_ = 1.15, *p* = 0.28, η^2^ = 0.005, 95% CI = [−1.239, 0.372]. However, the trend was that participants primed with individualism (mean = 4.16, SD = 1.60) were less willing to be added as a coauthor than people primed with collectivism (mean = 4.41, SD = 1.56).

The main effect of experience publishing was also not significant, *F*_(1, 219)_ = 0.02, *p* = 0.89, η^2^ = 0.00, 95% CI = [−0.639, 0.674]. However, graduate students (mean = 4.34, SD = 1.63) were more willing to be added as a co-author than undergraduates (mean = 4.18, SD = 1.43). There were no other main effects or interaction effects (Figure 4).

In sum, Study 5 found a causal relationship between individualism/collectivism and willingness to add an unmerited co-author. Putting people in an individualistic mindset made them less likely to grant unmerited authorship; putting people in a collectivistic mindset made them more likely to grant unmerited authorship.

To give a sense of whether the studies had adequate statistical power, we analyzed the *post-hoc* statistical power of all of the laboratory and survey studies using G^*^Power 3.1 (Faul et al., 2009). Study 2 had 66% power (*d* = 0.52). Study 3 had 99% power for adding a colleague as co-author (*d* = 2.60) and for being added by a colleague as co-author (*d* = 1.53). Study 4 had 99% power for adding a colleague as co-author (β = 0.27) and 18% power being added by a colleague as co-author (β = 0.11), which was not significant. Study 5 had 99% power adding a colleague as co-author (*d* = 0.57) and 27% power for being added by a colleague as co-author (*d* = −0.13).

In sum, the effect size for being added as a co-author was smaller than adding a colleague as a co-author. Thus, the studies generally did not have large enough samples to reliably detect an effect on this variable, and indeed the results were often not significant for this variable. However, for adding a colleague as a co-author, the effect size was larger, and power was high (average power = 91%).

### General discussion

Across five studies, we found evidence that collectivistic scientists tend to add more unmerited authors than individualistic scientists. We found that differences in unmerited authorship preferences between individualists and collectivists are broad in scope (between cultures and within cultures) and level (attitude, priming condition, behavioral intention, behavior implied in large author lists). The five studies reported in this article rely on different research methods: archival data analysis, online survey, lab studies, and experiments. Taken together, the data provide multi-source evidence in support of the hypothesis.

These findings intersect two fields: the cultural perspective on individualism/collectivism (Markus and Kitayama, 2010; Kitayama and Uskul, 2011) and scientific ethics (Marušic et al., 2011). The findings contribute to the individualism/collectivism literature by extending it to the behavior of scientists. This study also documents a cultural phenomenon at the group level, individual level, and causal level.

This is important given the fact that studies have documented that cultural differences at the group level do not have the same structure at the individual level (Na et al., 2010). For example, wealthy Americans are more likely to vote conservative (individual level), but wealthy states are more likely to vote liberal (group level; Gelman et al., 2008). In this study, the relationship between collectivism and unmerited authorship held at the individual level, group level, and in experimental priming.

As we accumulate more studies that test cultural phenomena at the individual level and the group level, we can start to fill in the picture. We can get an idea of how many phenomena work only at the group level and how many work at both the individual level and group level. We may even be able to build out theory explaining in broad terms which types of phenomena cross individual and group levels and why.

Finally, this paper contributes to calls for empirical research on cultural differences in inappropriate authorship (Street et al., 2010). This study provides at least a partial explanation for the previous finding that collectivistic nations have more reported authorship problems (Fetters and Elwyn, 1997; Marušic et al., 2011). This happens even though scientific committees have set standards to define scientific authorship.

Despite these standards, people may define contributions differently across cultures. For instance, people may consider contributions to include small favors such as providing a suggestion for how to interpret results. In individualistic cultures, that colleague would be excluded from co-authorship; in collectivistic cultures, that colleague might be included.

#### Implications for cultural psychology and moral decision-making

Individualism/collectivism was a hot topic in cultural psychology for many years, and hundreds of studies have been conducted on it. Psychologists have used three types of methods used to assess individualism/collectivism: self-report scales (Hofstede and Hofstede, 2001), implicit psychological tasks (Kitayama et al., 2009), and cultural products (Morling and Lamoreaux, 2008; Twenge et al., 2012). Most studies use just one type of outcome, which limits their validity. This study provides a more comprehensive picture because it uses multiple methods, including cultural products (Study 1), self-reports (Studies 2–4), and an experimental manipulation (Study 5).

This study also contributes to our understanding of cultural differences in moral decision-making and a mechanism behind those differences (Graham et al., 2011; van Leeuwen et al., 2012). This study found differences in how scientists weigh abstract ethical standards vs. relationship concerns. And it found that priming people with collectivism made them more likely to weight relational concerns over abstract standards.

This finding adds to previous findings that people in Eastern cultures weigh relationship concerns more heavily in their moral decisions (Miller and Bersoff, 1992; Graham et al., 2011). It also adds to a previous finding that priming people with collectivism makes them more likely to say they would pay a bribe (Mazar and Aggarwal, 2011). Future research can attempt to replicate the notion that priming collectivism can alter people's moral decisions. Further research can also look more deeply into the mechanism that priming collectivism influences. The bribery study found that collectivism lowered people's sense of responsibility for their actions (Mazar and Aggarwal, 2011, Study 2a). Does collectivism also change how people weight relationships? Does it change what people predict will be the social consequences of offending others (e.g., Yamagishi et al., 2008)? Future studies can parse test these mechanisms.

#### Implications for unmerited authorship

One interesting finding in this study was that Chinese students who had experience publishing were more likely to endorse unmerited authorship than students who had not yet published. This difference could be evidence that the experienced students have faced pressure to include unmerited authors and learned that it is acceptable. In other words, this may be evidence of acculturation. This also suggests that if we want to change authorship practices, it may be best to start educating scientists before they get to graduate school.

This study has several strong points: (a) a large cross-national sample; (b) multiple sources to measure unearned authorship practices; and (c) multi-level evidence. Nevertheless, it also has weaknesses that can be addressed in future research. For example, the mechanism between individualism/collectivism and unmerited authorship is unclear. One possibility is that collectivism pushes people to consider relationships when they make decisions about authorship. Another possibility is that collectivism is related to how people understand the meaning of “contribution.” Perhaps collectivism takes a broader perspective on contributions. Our studies cannot speak to this possibility, but future studies can test this.

Another limitation is that differences in people's willingness to be added as a coauthor showed non-significant trends in Study 4 and 5. We can think of two explanations for this. First, Studies 4 and 5 were within-culture comparisons, and within-culture differences tend to be smaller than between-culture differences (Heine et al., 2002). Study 3 compared two cultures and did find significant differences. Second, people are more likely to be biased toward themselves when they consider being added as a coauthor. This could decrease the amount of variation in the outcome measure (Honkaniemi and Feldt, 2008; Zettler et al., 2015).

This study focused on the natural sciences. Thus, it could be expanded by including social scientists. Some social sciences have different authorship practices than other fields (Marušic et al., 2011). For example, management and economic studies use alphabetical order of co-authors as a norm to address authorship problems (Frandsen and Nicolaisen, 2010). We presume the link between individualism/collectivism and unmerited authorship extends to the social sciences as well, but this requires further empirical evidence.

We focused on individualism and collectivism in this study, but there may be other factors that differ across cultures that could explain the differences. For example, one reviewer of this paper suggested that students in some cultures may receive less training on authorship practices. Factors like this may reflect underlying differences in collectivism, or they may be independent third factors.

## Conclusion

Unmerited authorship can have negative effects on science, both in the short term and long term (O'Brien et al., 2009; Wager, 2009). Through five studies, we found evidence that this misconduct is related to culture. Collectivist scientists are more willing to grant unmerited authorship credit than individualistic scientists at both the cultural and individual level. With an improved understanding of one of the causes of this problem, we are in a better position to know how to go about reducing it.

## Ethics statement

This study was carried out in accordance with the recommendations of Institutional Review Board of the Institute of Psychology, Chinese Academy of Sciences with written informed consent from all subjects. All subjects gave written informed consent in accordance with the Declaration of Helsinki. The protocol was approved by the Institutional Review Board of the Institute of Psychology, Chinese Academy of Sciences.

## Author contributions

XR: Study design and manuscript writing. HS, KL, XD: Data collection and Statistics analysis. ZO: Data collection. TT: Manuscript writing.

## Funding

The research was supported by a grant from Ministry of Science and Technology of China (2009FY110100) and Open Research Fund of the Key Laboratory of Behavioral Science, Institute of Psychology, Chinese Academy of Sciences. The assistance of Michael M. Varnum in conducting this research is gratefully acknowledged.

### Conflict of interest statement

The authors declare that the research was conducted in the absence of any commercial or financial relationships that could be construed as a potential conflict of interest.

## Footnotes

^1^We also ran an ANOVA including these 24 scientists who were born in Eastern cultures and worked in the West were included. The results also showed cultural differences in co-author practices, *F* = 6.32, *p* = 0.002. *Post-hoc* analysis using least squares differences revealed that both Western scientists (mean difference = 0.27, *SE* = 0.08, *p* = 0.001) and Eastern-born-Western-working scientists (mean difference = 0.32, *SE* = 0.18, *p* = 0.007) are more reluctant to add unmerited coworker on list than those from eastern. Differences between Western scientists and Eastern-born-Western-working scientists were not significant (mean difference = 0.04, *SE* = 0.09, *p* = 0.64). This suggests that living in a Western culture pushes scientists to adopt attitudes more common in Western cultures.

^2^we did a correlation and mediation analysis to see if there's correlation between individualism and attitudes to unmerited authorship. Individualism was negatively correlated with adding a colleague as a coauthor, *r*_(32)_ = −0.49, *p* < 0.01, and negatively correlated with being added by a colleague as a coauthor *r*_(32)_ = −0.26, *p* = 0.15. The latter correlation was not significant. For the correlation analyses done separately by culture, there were no correlations between individualism and unmerited authorship (adding a colleague as coauthor and being added by a colleague as a coauthor). We proceeded to do a mediation/moderation analysis, and the results showed that individualism does not mediate or moderate the relationship between culture and unmerited authorship (adding a colleague as coauthor and being added by a colleague as a coauthor). It should be noted that we cannot go further to support or reject the hypothesis in study 3 due to the small sample size. The only thing that can be confirmed is that there are cultural differences on unmerited authorship between Danish and Chinese.
