# Supplementary material for: Culture and Unmerited Authorship Credit: Who Wants It and Why?
Source: Front Psychol. 2016 Dec 27;7:2017. doi: 10.3389/fpsyg.2016.02017 (PMC5186795; doi:10.3389/fpsyg.2016.02017)
Supplement: Supplementary file 1 [file Table1.docx]

Supplementary Material

Culture and Unmerited Authorship Credit: Who Wants It and Why?

Author: Xiaopeng Ren , Hong Su, Kewen Lu, Jiang Xu, Zhengzheng Ouyang, Thomas Talhelm

*** Correspondence: renxp@psych.ac.cn**

# Supplementary Tables

Table 1A. Six items developed to measure co-author practices

| Item 1 | I have included a colleague as a co-author who made no contribution to the manuscript, but with whom I have a personal relationship. |
| --- | --- |
| Item 2 | I have included a colleague as a co-author who made no contribution to that particular manuscript, but with whom I had collaborated in the past and planned to work with in the future. |
| Item 3 | I have included a colleague as a co-author who made a minimal contribution to the manuscript, but with whom I had collaborated in the past and planned to work with in the future. |
| Item 4 | I have been included as a co-author on a manuscript to which I made no contribution, but with whose author I have a personal relationship. |
| Item 5 | I have been included as a co-author on a manuscript to which I made no contribution, but with whose author I have collaborated in the past and plan to work with in the future. |
| Item 6 | I have been included as a co-author on a manuscript to which I made a minimal contribution, but with whose author I have collaborated in the past and plan to work with in the future. |
